# Foamy Virus Vectors for HIV Gene Therapy

**DOI:** 10.3390/v5102585

**Published:** 2013-10-22

**Authors:** Miles E. Olszko, Grant D. Trobridge

**Affiliations:** 1 Department of Pharmaceutical Sciences, Washington State University, Pullman, WA 99164, USA; E-Mail: grant.trobridge@wsu.edu; 2 School of Molecular Biosciences, Washington State University, Pullman, WA 99164, USA

**Keywords:** foamy virus, lentivirus, retrovirus, vector, gene therapy, HIV

## Abstract

Highly active antiretroviral therapy (HAART) has vastly improved outcomes for patients infected with HIV, yet it is a lifelong regimen that is expensive and has significant side effects. Retroviral gene therapy is a promising alternative treatment for HIV/AIDS; however, inefficient gene delivery to hematopoietic stem cells (HSCs) has so far limited the efficacy of this approach. Foamy virus (FV) vectors are derived from non-pathogenic viruses that are not endemic to the human population. FV vectors have been used to deliver HIV-inhibiting transgenes to human HSCs, and they have several advantages relative to other retroviral vectors. These include an attractive safety profile, broad tropism, a large transgene capacity, and the ability to persist in quiescent cells. In addition, the titers of FV vectors are not reduced by anti-HIV transgenes that affect the production of lentivirus (LV) vectors. Thus FV vectors are very promising for anti-HIV gene therapy. This review covers the advantages of FV vectors and describes their preclinical development for anti-HIV gene therapy.

## 1. HIV and HAART: Limitations of the Current Standard of Care

HIV infection remains a global health crisis. There were an estimated 34 million cases worldwide in 2010 [1], and there is still no effective vaccine. The current standard of care for HIV infection is highly active antiretroviral therapy (HAART), a treatment strategy that uses cocktails of antiretroviral drugs to control HIV replication and to delay disease progression to AIDS. HAART has greatly improved outcomes for individuals living with HIV [2,3]. It efficiently inhibits viral replication and is highly effective at suppressing viral loads. 

However, there are several limitations to HAART that make the development of novel therapies including gene therapy a high priority. So far, HAART therapy has not been able to target latent proviruses residing in long-lived cellular reservoirs [4]. These reservoirs appear to be stable [5], and they are thought to be the source of the low level viremia that is typically observed over the lifetime of an HIV patient on HAART [4,5]. Lack of adherence to HAART regimens and the possibility of pharmacological sanctuary sites [6,7,8] are also major concerns as they may accelerate the generation of drug resistant HIV mutants [7,9,10]. Because infectious viral particles generally persist at a baseline level in the blood under a HAART regimen [11,12], interruption of treatment leads to viral rebound [13]. HAART is a lifelong treatment and is associated with toxicity that may have significant effects on longevity and quality of life [14,15,16]. In addition, HAART is expensive. The annual cost for HAART therapy in the U.S was $13,000 per person per year for antiretrovirals alone, based on 2006 data [17]. Although HAART has served as the cornerstone of anti-HIV therapeutics for nearly two decades, it has limitations that justify the exploration of alternative treatments.

## 2. HIV Gene Therapy

Retroviral gene therapy is a potential alternative to HAART. Under this strategy, hematopoietic cells are harvested from a patient and gene-modified *ex vivo* by transduction with a retroviral vector. The transduced cells are then reintroduced to the patient’s body. Both hematopoietic stem cells (HSCs) and CD4 T cells have been explored as cell targets. A major advantage of HSC gene therapy is that HSCs produce all the mature cells that are infected by HIV including CD4 cells, macrophages and dendritic cells. HSCs carrying anti-HIV transgenes would persist over the lifetime of the patient, continually producing differentiated daughter cells that are protected against HIV infection. A major advantage of this approach is that it would be a one-time procedure and, thus, eliminate the need for patients to comply with complicated and expensive HAART treatment regimens.

Early trials have demonstrated the efficacy of anti-HIV transgenes delivered by retroviral gene therapy. However, use of these therapies has been complicated with low levels of gene marking [18,19]. It is clear that the efficiency of gene transfer of anti-HIV transgenes must be improved. Another challenge for gene therapy is safety. Following the development of leukemia in SCID-X1 patients who received HSC gene therapy [20,21,22,23], major efforts have gone into better understanding the risks of different vector systems and into improving the safety of retroviral vectors. Safe vector systems will be an important consideration to move HIV gene therapy to a front line treatment for HIV/AIDS.

## 3. Retroviral Vectors for HIV Gene Therapy

Retroviruses have been the delivery vector of choice for HIV gene therapy clinical trials due to their ability to efficiently integrate, allowing for efficient transmission of anti-HIV transgenes to all daughter cells. Current retroviral vector systems derived from lentivirus (LV), foamy virus (FV) and gammaretrovirus (GV) are replication-incompetent, and have been engineered with several safety features. During vector production the viral helper functions are physically separated from the retroviral vector and provided on separate helper plasmids. Currently used systems have advanced to the point that contaminating replication-competent viruses are not generated. Advanced retroviral vectors have been engineered to be self-inactivating (SIN) [24]. SIN vectors are replication-incompetent due to the deletion of the viral promoter and enhancers in the U3 region of the 3′ vector long terminal repeat (LTR). This deletion is copied to the 5' LTR during reverse transcription, resulting in deletions in both LTRs of the integrated provirus. SIN vectors are less likely to activate nearby genes than non-SIN vectors [25].

## 4. Limitations of LV Vectors

**Figure 1:**
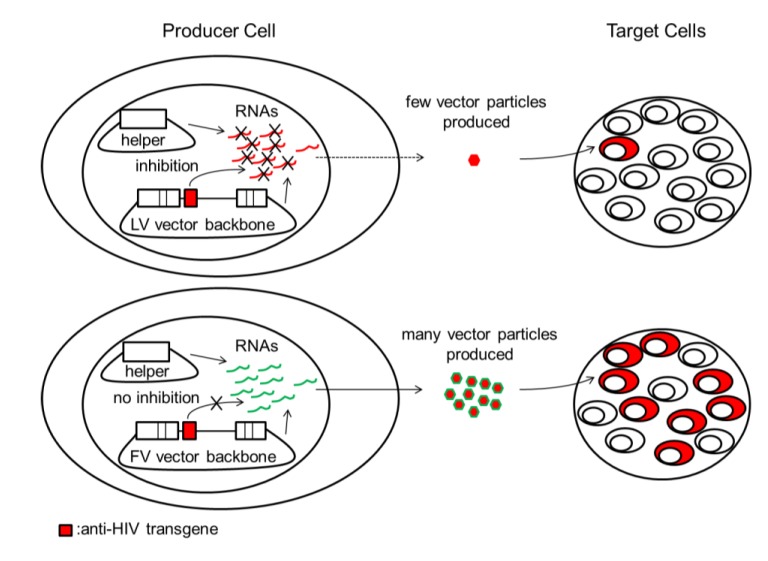
Inhibition of LV vector production by anti-HIV transgenes can lead to low titers and poor transduction efficiency in target cells. HIV-based LV vectors and HIV share identical nucleotide sequences and proteins. During LV vector production, LV vector backbones and LV helper plasmids are cotransfected into producer cells to produce LV vector virions for infecting target cells. LV vector plasmids and/or LV helper plasmids and their respective RNAs can be targeted by some anti-HIV transgenes such as short hairpin (sh)RNAs (red box). This can result in a reduction in the number of vector particles produced, leading to inefficient transduction of target cells by low titer LV vector. FV vector plasmids, FV helper plasmids, and their respective RNAs are not affected because FV vectors do not share significant sequence identity with HIV. HIV/LV vector components and anti-HIV transgenes are indicated in red. FV vector components are indicated in green. LV, lentivirus; FV, foamy virus.

Much of the recent focus in HIV gene therapy has been directed towards efforts utilizing LV vectors derived from HIV-1. These vectors are widely used in part because of their ability to efficiently transduce non-dividing cells. However, the use of LV vectors is complicated by the fact that HIV-1 based vectors have nucleotide sequences and also some proteins of the HIV virus itself. The titers of LV vectors can be severely suppressed by the expression of anti-HIV transgenes that target functions that are shared by LV vectors and HIV [26,27,28,29,30] (Figure 1). For *in vitro* studies using transformed cell lines as models for protection, high titer vector preparations are not needed to efficiently deliver anti-HIV transgenes. However, vector titer is a critically important consideration for clinical studies where low anti-HIV vector titers can severely reduce gene transfer efficiency to quiescent HSCs. While some investigators have been able to compensate for inhibited vector production on a case-by-case basis [26,28,29,30], the use of LV vectors for anti-HIV gene therapy can complicate vector design. It may even preclude the use of some anti-HIV transgenes, or some transgene combinations if they synergize to reduce anti-HIV LV vector titers. Because even SIN LV vectors have residual transcriptional activity from their LTRs [31], another potential problem with using LV vectors is that integrated proviruses could recombine with and/or be mobilized by HIV. So far, however, the risk appears to be small for SIN LV vectors [29]. One approach to address the problems with HIV-1-based vectors is the use of LV vectors that are not based on HIV-1. HIV-2-based vectors have been used in anti-HIV gene therapy, but much of the work has been done on vectors that are mobilized by HIV-1 [32,33]. Other LV vectors such as feline immunodeficiency virus (FIV) [34] and equine infectious anemia virus (EIAV) [35] vectors have also been developed. However, EIAV vectors do not transduce human HSCs as efficiently as second generation HIV-1-based vectors [36], and inefficient transgene expression from FIV vectors in human hematopoietic cells has been reported [37].

## 5. FV Vectors

**Figure 2:**
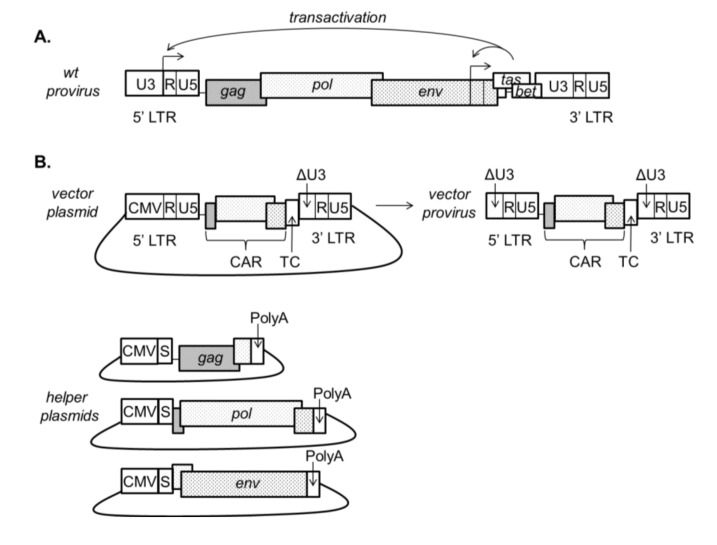
(**A**) Wild type FV provirus; (**B**) A third generation minimal FV vector backbone and helper plasmids. The vector is shown as it would appear in a plasmid for vector production and as an integrated provirus. Third generation FV vectors include a deletion of the transcriptional transactivator, *tas* (Previously known as *bel-1*), which acts at the viral LTR in wild type FV. These vectors are SIN due to the removal of the transactivator *tas* and have a deletion encompassing the TATA box and enhancers in the U3 region of the 3' LTR of the vector plasmid (ΔU3 in figure). This deletion is copied to the 5' end of the viral genome during reverse transcription, resulting in the silencing of both LTRs in the integrated provirus. In contrast to LV vector systems, *gag* and *pol* in FV vector systems are translated from separate mRNAs, and *gag*, *pol* and *env* genes are provided in *trans* on three separate helper plasmids. *Cis*-acting regions (CAR) remain on the vector backbone. Abbreviations: CAR, *cis*-acting region; CMV, cytomegalovirus promoter; LTR, long terminal repeat; S, SV40 intron; TC, transgene cassette; Poly A, poly adenylation site.

FV vectors have several important advantages for HIV gene therapy. The FVs, or spumaviruses, are ancient retroviruses that have undergone extensive co-evolution with their natural hosts [38,39]. They are endemic in non-human primates (NHPs) and other mammals, but have not been detected in humans except in cases of benign zoonosis. These zoonotic infections are usually acquired through hunting or occupational exposure to NHPs [40,41]. FVs have not been observed to be transmitted between humans, and unlike the LVs, FVs do not cause disease in their hosts. FV vectors have a broad cell tropism [42]. In addition, methods for pseudotyping FV vectors have been described [43]. In terms of genome size, FVs are among the largest of the retroviruses [44] and FV vectors are capable of packaging large transgenes. In one study, a vector was generated with a 9.2 kb insert, bringing the total vector length close to the parent virus size. This vector could be produced at approximately one third the titer of a vector with a 2.4 kb insert [45]. FV vectors also have an attractive safety profile relative to GV vectors and LV vectors (See below). FVs are unusual among retroviruses in that reverse transcription frequently takes place in the cell producing virions, rather than prior to integration in the infected cell. As a consequence, unlike other retroviruses, many of the infectious particles of FVs contain dsDNA genomes [46]. While mitosis is required for FV vector transduction, FV vectors form a highly stable transduction intermediate in quiescent cells [47] and this may explain their efficient transduction of HSCs. Transduction efficiencies of FV vectors in HSCs are comparable to those of LV vectors [48]. A stable FV vector transduction intermediate may also explain why very short *ex vivo* transduction protocols can be used for gene delivery to HSCs in a large animal model [49]. This is important because protocols with extended *ex vivo* culture times reduce engraftment [50]. Advanced, third generation SIN vectors [45] (Figure 2) based on the prototypic FV and other FVs have shown great promise in preclinical studies.

## 6. Vector Genotoxicity

Integrating retroviral vectors are insertional mutagens that modify the genome. Hence, they pose a risk of oncogenesis when used for gene therapy. Clonal expansion and leukemia have occurred in gene therapy clinical trials as a result of vector-mediated dysregulation of nearby genes [20,21,22,23,51,52]. As a result, improving vector safety is a major priority for the field of gene therapy. Integrating vectors differ in their preferences for insertion sites within the host genome [53]. They also differ in their likelihood to dysregulate nearby genes [54]. Together, these factors influence vector genotoxicity.

### 6.1. Retroviral Vector Integration Profiles

Among GV vectors, LV vectors, and FV vectors, LV vectors have the greatest preference for integrating within genes. FV vectors are approximately twofold less likely than LV vectors to integrate within genes, and are less likely than GV vectors to integrate near transcription start sites [53]. While these results are encouraging for the use of FV vectors in the clinic, clearly there is still room for improvement. For example, modifying the FV integration profile to reduce the frequency of integration near proto-oncogenes may improve safety. Factors influencing vector integration profiles are not well understood, but they are known to include both the state of target chromatin and the effect of chromatin tethering functions mediated by interactions between viral and host proteins [55,56,57]. Some success in retargeting LV and GV vectors has been reported [56,58,59], suggesting that it may also be possible to retarget FV vectors to improve safety.

### 6.2. Dysregulation of Neighboring Genes

Vector-mediated dysregulation of neighboring genes occurs by several different mechanisms including enhancer-mediated activation, truncation of cellular transcripts, and read through transcription. For a review see [60]. While SIN LV vectors appear less likely than GV vectors to dysregulate nearby genes through enhancer-mediated activation, there is evidence that LV vector proviruses allow significant read-through transcription and generate chimeric transcripts [54,61]. This can contribute to clonal expansion and oncogenic potential [62]. FV vectors are more resistant to read-through transcription than LV and GV vectors, presumably in part due to efficient polyadenylation although other factors may be involved [54]. This can reduce the potential to activate nearby genes and is an important safety advantage for FV vectors.

### 6.3. Vector Design to Reduce Genotoxicity

The use of insulator elements [63] and elimination of potential splice sites can improve safety. In addition, efficient polyadenylation signals in vector LTRs [64] can reduce the potential for genotoxicity. The use of weaker housekeeping promoters, such as the elongation factor 1α promoter, rather than strong viral promoters such as the spleen focus forming virus (SFFV) promoter to drive the transgene cassette can decrease the probability of vector-mediated dysregulation of neighboring genes [65]. However, some vector modifications may reduce efficacy. For example, using weaker promoters can result in reduced transgene expression. Thus, efforts to improve safety must be balanced with the need for clinical efficacy.

## 7. FV Vector HSC Gene Therapy Models

Encouraging data obtained from preclinical studies with FV vectors have increased interest in FV vectors for HSC gene therapy. FV vectors have been investigated in several animal models, notably the non-obese diabetic/severe combined immunodeficiency (NOD-SCID) and NOD-SCID IL2Rγ^null^ (NSG) mouse, and also the dog large animal model. Xenotransplantation of FV transduced human CD34 cells has been demonstrated in immunodeficient mouse strains such as the NOD-SCID and NSG models [27,66,67]. Gene marking has been observed in multiple hematopoietic lineages, indicating the potential of FV anti-HIV vectors to protect mature cells in the myeloid and lymphoid lineages from HIV infection. While mouse models have provided important preclinical data for the use of FV vectors for HSC gene therapy, the short lifespans of mice and differences in HSC characteristics in mice and primates impose some limitations [68,69]. Large animal models have better predicted clinical efficacy of HSC gene therapies and also allow for studies of long term repopulating cells. The dog large animal model has several advantages. Dogs are easily cared for, they reproduce quickly, their HSC physiology is similar to that of humans [70], and they can be used to model several human hematopoietic diseases [71,72,73]. FV vectors efficiently transduce canine long-term repopulating HSCs [49]. In a direct comparison with LV vectors, FV vectors transduced canine long term repopulating HSCs at similar efficiencies to LV vectors [48]. Canine leukocyte adhesion deficiency (CLAD) [74] and pyruvate kinase deficiency [75] have been corrected in dogs using FV vector HSC gene therapy. CLAD dogs receiving FV vector HSC gene therapy did not develop leukemia as a result of vector mediated oncogenesis in the years following infusion [76]. This large animal data strongly supports the safety of FV vector gene therapy.

## 8. FV Vector Anti-HIV Studies

A number of anti-HIV transgenes and transgene combinations have been explored for use in FV vector-mediated anti-HIV gene therapy [27,77,78,79]. These are summarized in Table 1. 

### 8.1. *In Vitro* Studies

An FV vector with a single shRNA targeting viral *rev/env* was used to inhibit simian immunodeficiency virus, a close relative of HIV, in *in-vitro* challenge assays. Results were encouraging, with inhibition of viral replication reaching 68%–80% [77]. However, single RNAi therapies against HIV are of limited use due to the ability of HIV to escape inhibition through mutation [80]. RNAi therapies are less vulnerable to viral escape when two or more sequences are expressed in combination, or when RNAi is expressed together with other classes of anti-HIV transgenes. Park *et al*. reported [78] using FV vectors to deliver anti-HIV transgene cassettes under the control of either a cytomegalovirus (CMV) promoter or a minimal heat shock promoter (Hsp), which is activated in the presence of HIV Tat. Transgenes under the control of the Hsp were expressed conditionally in the presence of HIV Tat through interaction of Hsp with TAR recruited proteins at an upstream partial HIV LTR, while transgenes under the control of the CMV promoter were constitutively expressed. An anti-HIV miRNA cassette targeting HIV Rev (R) and the HIV LTR (L2) was highly effective under the control of either promoter, inhibiting HIV replication by >98% in a challenge assay. The investigators also tested the effectiveness of an anti-*rev* miRNA cassette under the control of Hsp, as well as the antiviral activity of the TAR expressed without any additional transgenes. The anti-*rev* miRNA cassette under the control of Hsp was found to inhibit HIV replication by >98% when challenged with HIV. The LTR expressing only TAR inhibited HIV replication to the same degree. The authors speculated that a TAR miRNA processed from the vector might have been responsible for the inhibitory effect.

**Table 1:** Anti-HIV Transgenes in FV Vectors. C46: membrane associated HIV fusion inhibitor; CMV: cytomegalovirus immediate early promoter; LTR: long terminal repeat; H1: human H1 RNA promoter; Hsp: heat shock promoter; L2R: LTR + *rev* miRNA; MSCV: murine stem cell virus promoter; PGK: phosphoglycerate kinase promoter; R2: SIV rev shRNA; R5: CCR5 shRNA; RevM10: dominant negative Rev; SFFV: spleen focus forming virus promoter; Sh1: anti *tat/rev* shRNA; SHIV: simian-human immunodeficiency virus; SI: *tat/rev* shRNA; SII: *tat/rev* shRNA; SIV: simian immunodeficiency virus; TAR: HIV trans-activation response element; U6: human U6 small nuclear RNA Pol III promoter.

Transgene	Description	Efficacy	Promoter	Assay	Publication
R2	SIV *rev* + *env* shRNA	68%–80% inhibition of viral replication	U6	SIV challenge, CEMx174 cell line	Park *et al*. 2005 [77]
L2R	HIV LTR + *rev* miRNA cassette	>98% inhibition of viral replication	CMV	HIV challenge, U87.CD4.CXCR4 cell line	Park *et al*. 2009 [78]
TAR + L2R	Tat inducible HIV LTR + *rev* miRNA cassette + TAR	>98% inhibition of viral replication	Tat inducible LTR-Hsp fusion		
TAR + R	Tat inducible *rev* miRNA cassette + TAR	>98% inhibition of viral replication	Tat-inducible LTR-Hsp fusion		
TAR	TAR	>98% inhibition of viral replication	LTR		
Sh1	anti *tat*/*rev* shRNA	4 log reduction of viral replication	U6	HIV challenge, CD34-derived macrophages	Taylor *et al*. 2008 [79]
C46	membrane associated fusion inhibitor	4 log reduction of viral replication	MSCV		
Sh1 + C46 + RevM10	*tat*/*rev* shRNA + membrane-associated fusion inhibitor + dominant negative Rev	significantly increased relative to C46 alone	U6, MSCV, PGK	HIV challenge of protected and unprotected cells in CEMx174 cell line	
C46	membrane associated fusion inhibitor	5.2-fold increase in cell survival +3.1-fold decrease in HIV p24/cell	MSCV		
		4 log reduction of viral replication	SFFV	SHIV challenge, CEM.NKR-CCR5 lymphocytes	Kiem *et al*. 2010 [27]
		15–20 fold reduction of viral replication	SFFV	SHIV or HIV single viral cycle challenge, MAGI-CCR5 cell line	
SI + C46	*tat*/*rev* shRNA + membrane associated fusion inhibitor	5 fold reduction of viral replication	U6, SFFV		
SII + SI + R5 + C46	two *tat*/*rev* shRNAs + CCR5 shRNA + membrane associated fusion inhibitor	23 fold reduction of viral replication	H1, SFFV		
		4 log reduction of viral replication		SHIV challenge, CEM.NKR-CCR5 lymphocytes	
SI + C46	*tat*/*rev* shRNA + C46	4 log reduction of viral replication	U6, SFFV		
SII + SI + R5	two *tat*/*rev* shRNAs + CCR5 shRNA	180 fold reduction of viral replication	H1		

Taylor *et al*. investigated FV vectors with an anti-*tat/rev* shRNA, a dominant negative mutant of HIV *rev*, and a membrane-associated HIV fusion inhibitor (C46) [79]. Both the anti-*tat/rev* shRNA and C46 potently inhibited viral replication in CD34-derived macrophages. When a challenge was performed on a mixture of gene-modified and also unprotected CEMx174 cells, improved survival and reduction of viral replication was observed. A vector expressing all three anti-HIV transgenes offered significantly better protection than C46 expressed alone. Finally, the relative effectiveness of each transgene and also a combination of all three transgenes was compared in a competitive challenge assay. Cells were transduced with FV vectors expressing each transgene individually, or all three. These FV-transduced cells were then combined in equal proportion and challenged with HIV. The ratio of cells expressing C46 or all three transgenes increased with time relative to cells expressing the *rev* mutant or the shRNA. This again suggested that C46 and the triple combination cassette were more effective at inhibiting HIV infection than the mutant *rev* or the shRNA expressed alone [79].

### 8.2. *In Vivo* Selection of Human SCID Repopulating Cells

The P140K mutant of the methylguanine methyltransferase gene (MGMTP140K) can be included in retroviral vectors to allow selection of transduced cells *in vivo*. The gene product of wild-type MGMT repairs alkylated guanine bases that are induced by chemotherapy drugs such as bis-chloroethyl nitrosourea (BCNU). Wild-type MGMT can be deactivated by guanine analogs such as O6-benzylguanine (O6BG) [81]; however, MGMTP140K is highly resistant to deactivation by this compound [82]. Administration of O6BG and BCNU to an animal engrafted with hematopoietic cells transduced with a vector expressing MGMTP140K kills unprotected (untransduced) cells. The result is the enrichment of cells that express MGMTP140K and the anti-HIV cassette. Importantly, this type of selection allows expansion of long term repopulating hematopoietic cells *in vivo* [83].

A study in the NSG mouse model demonstrated efficient engraftment and expansion of human CD34 cells transduced with an FV vector that incorporated an anti-HIV transgene cassette and MGMTP140K [27]. The anti-HIV cassette encoded a combination of C46, two shRNAs against HIV-1 *rev* and *tat*, and a shRNA against CCR5, a macrophage-tropic HIV-1 coreceptor. Of several transgene cassettes tested in a single cycle *in vitro* assay, this combination was found to most potently inhibit both HIV and simian-human immunodeficiency virus (SHIV), a chimeric human-simian immunodeficiency virus used to model HIV infection in primates. The inclusion of the MGMTP140K transgene allowed for *in vivo* selection of gene modified cells using O6BG and BCNU. This allowed for a significant increase in the percentage of marked cells in the bone marrow of NSG mice. Importantly, the vector used in this study could be produced at a titer of 3.8 × 10^7^ transducing units∙mL^−1^, sufficient for clinical studies. 

### 8.3. Anti-HIV shRNAs Inhibit LV but Not FV Vector Production

The titers of LV and FV vectors expressing the same anti-HIV transgene cassette that included a *tat/rev* shRNA have also been directly compared [27]. The FV vector titer was not affected compared to a control vector, however a LV vector was reduced in titer 150-fold. This is consistent with observations that transgenes targeting *rev* or its gene product reduce LV vector titers by inhibiting expression of Rev from LV helper plasmids or LV vector backbones during vector production [28,29].

## 9. Conclusions

FV vectors offer several important advantages over other retroviral vectors for HIV gene therapy. They can package large transgene cassettes and they have a desirable safety profile. Although FV vectors are potentially suited for broad use in HSC gene therapy, they may prove particularly useful in the treatment of HIV/AIDS. Lower sequence and functional homology between FV vectors and HIV reduces the probability of recombination with and/or mobilization by endogenous HIV, and FV vectors avoid the reduced titers observed in LV vectors carrying some anti-HIV transgenes. FV vector platforms may therefore better avoid complications in vector design and more efficiently produce safer, high titer anti-HIV-transgene containing vector than LV vector systems. FV anti-HIV vectors have been developed by several groups and have shown great promise in preclinical studies. Additional studies to improve FV vector safety by modifying vector components, and continued development of FV vectors with potent anti-HIV transgene combinations should lead to FV vectors with excellent clinical potential.
